# The Serine-Threonine Protein Kinase PAK4 Is Dispensable in Zebrafish: Identification of a Morpholino-Generated Pseudophenotype

**DOI:** 10.1371/journal.pone.0100268

**Published:** 2014-06-19

**Authors:** Sheran H. W. Law, Thomas D. Sargent

**Affiliations:** Section on Vertebrate Development, Program on Genomics of Differentiation, Eunice Kennedy Shriver National Institute of Child Health and Human Development, National Institutes of Health, Bethesda, Maryland, United States of America; Texas A&M University, United States of America

## Abstract

TALEN-based inactivation of the zebrafish *pak4* gene resulted in embryos and adult fish that appear normal and fertile. This is in contrast to our previously published studies which were based on the use of antisense morpholino oligonucleotides (MOs). We have excluded potential explanations such as gene duplication, alternate splicing, cryptic initiation of translation, and translation-independent RNA function. Our conclusion is that *pak4* is dispensable in zebrafish, and that even when corroborated by robust controls, such as RNA rescue, MOs may elicit misleading pseudophenotypes that do not correspond to results obtained by genetic mutations, and should thus be used with caution.

## Introduction

The optical clarity, rapid, in vitro embryonic development and relatively short generation time of zebrafish (*Danio rerio*) have led to the extensive use of this species for the characterization of gene function in vertebrate development. Classical genetic screens of mutagenized zebrafish have been highly successful in the identification of genes required for primary embryonic axis formation and numerous aspects of tissue and tissue differentiation [1], [2], yielding important insights with broad significance for developmental biology and human health. However, the majority of gene function studies in zebrafish have been based on the use of antisense oligonucleotides with degradation-resistant morpholino phosphorodiamidate backbones (MOs). These have been shown to interact specifically with complementary RNA targets, resulting in inhibition of translation, RNA splicing, and other steps in post-transcriptional gene regulation (e.g Lim et al., 2012 [3]). However, it has become evident that in addition to specific inhibition of the cognate target genes, MOs can also have side effects. Ekker and Larson [4] reported that 15–20% of tested MOs show non-specific toxicity on developing embryos, with the most common artifact being induction of apoptosis in one or more tissues, frequently but not exclusively in the brain (reviewed by Eisen and Smith, 2008 [5]). Ekker and colleagues showed this was due at least in part to activated expression of an N-terminal truncated isoform of the transcription factor p53, and that the MO-induced apoptosis artifact could be suppressed by adding an antisense p53 MO to the injection cocktail [6]. In addition to cell death, MO-induced p53 can also elicit patterned gene expression via activation of pro-apoptotic factors, that can be mistaken for developmental regulation [7]. In many cases, legitimate effects by MOs on gene expression have been distinguished from artifacts by using a rescue control in which a synthetic mRNA, engineered to lack the MO recognition sequence, is co-injected. If the MO-induced phenotype can be reverted to the control pattern, the knockdown has been deemed to result specifically from the loss of target gene expression. We used MOs, in combination with both of these types of controls, to analyze the developmental function of the serine/threonine protein kinase PAK4 in zebrafish [8].

PAK4 is one of six related proteins that mediate signal transduction via Rho-class GTPases to control a plethora of biological processes, including cytoskeletal dynamics, cell polarity and migration [9]. The family can be subdivided into two groups, PAK1-3, which are tightly regulated by interaction with GTPases, and PAK4-6, which bind specifically to GTPases, but have kinase activity that does not depend on this binding. In addition, some functions of the Group II PAKs may be independent of protein kinase activity [10], [11]. In mouse, loss of *pak4* function via gene targeting results in a complex lethal phenotype including disruption of placental and epiblast vasculature, heart failure, defective neurons and folding of caudal neural tube. Some aspects of this phenotype might be secondary effects of vascular failure [12], [13]. The loss of function phenotype we observed in zebrafish is also lethal, but differs in many respects from that found in mouse. The zebrafish egg contains high levels of *pak4* mRNA, but PAK4 protein is not detectable until the high stage of cleavage, approximately three hours after fertilization. Consistent with this maternal mRNA storage, we found that loss of *pak4* expression in zebrafish behaved as a maternal effect: disruption of zygotic expression alone, via splice-inhibitory MOs, had no effect on development, whereas inhibition of translation using a MO targeting the 5′-end of the open reading frame, alone or in conjunction with splice MOs, resulted in complex defects on the formation of primary myelopoietic cells, vasculature, and somite formation. All of these defects were shown to be independent of the p53-mediated interference noted above, and could be efficiently rescued by the injection of synthetic mRNA lacking the MO target sequence [8].

Because of the uncertainties associated with MOs, and to provide a genetically defined platform for further analysis of *pak4* function in zebrafish, we used TALEN-based gene targeting to establish germ-line loss-of-function mutations. TALENs (transcription activator-like effector nucleases) are recombinant proteins based on the TALE transcription factors of *Xanthomonas* bacteria. These have been used to generate site-specific double strand breaks in the genomes of several metazoan species, including zebrafish. These breaks are usually repaired by non-homologous end joining, which is error prone, frequently leading to small insertions or deletions (indels) at the repair site. When such indels result in a translational frame shift, loss of function for the encoded protein may be inferred. This strategy was used to generate frame shifts just downstream from the N-terminal methionine codon in the *pak4* transcript, resulting in complete loss of this protein. In contrast to the results with MOs, *pak4* null zebrafish were found to be normal and fertile, in both F_2_ and F_3_ homozygous and compound heterozygous mutant individuals. We conclude that *pak4* function is dispensable in zebrafish, and that the antisense MO approach, even with robust RNA rescue controls, may be subject to misleading results.

## Materials and Methods

### Animals and Ethics Statement

Zebrafish of EK background were used in this study. Embryo handling and care was carried out using standard procedures [14]. All experiments using zebrafish were performed according to the NICHD IACUC-approved animal study protocol # 12-039. This ASP is not used for other projects.

### Design and Generation of TALENs

**Figure 1:**
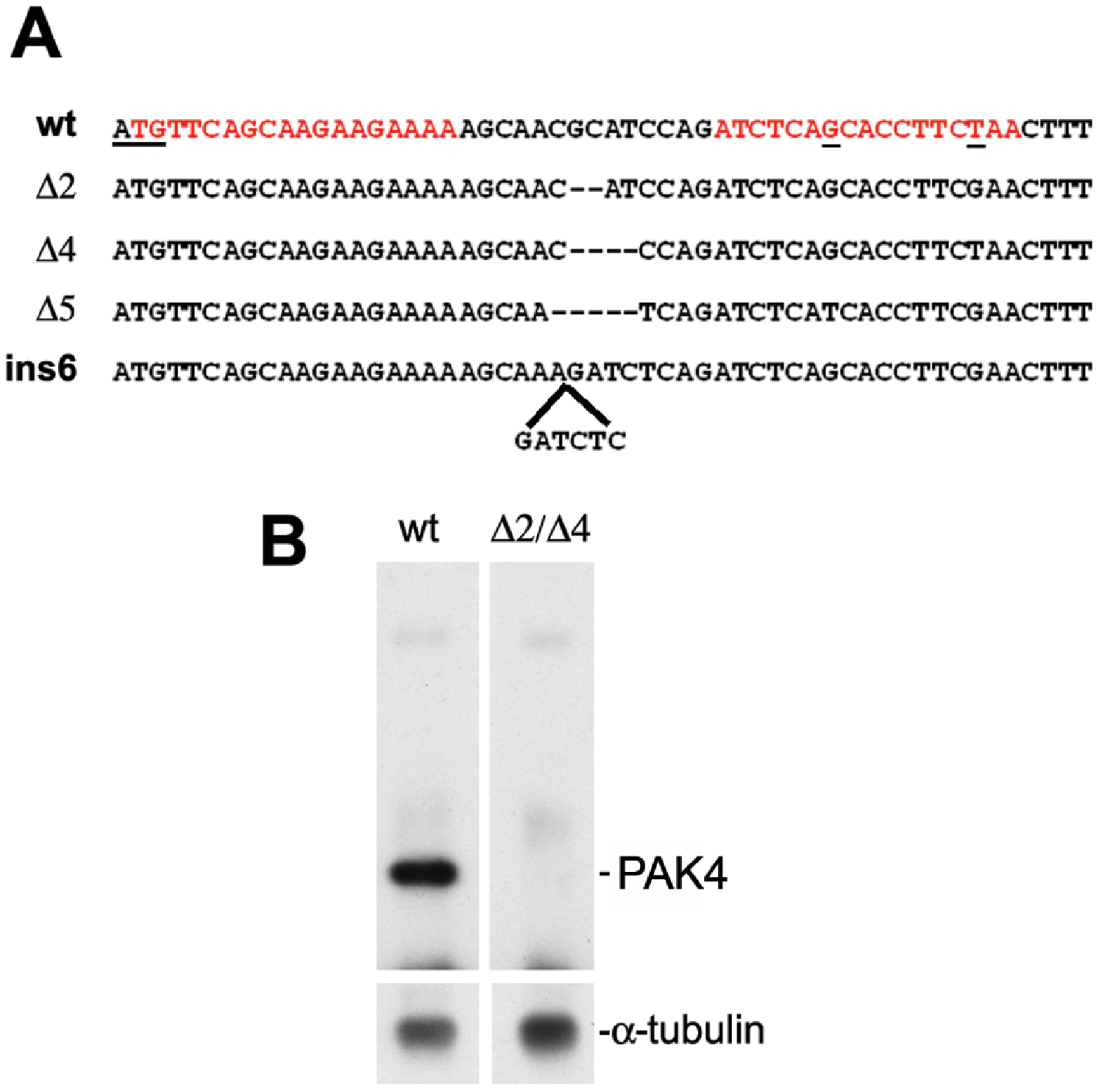
TALEN design and results of indel mutations in zebrafish *pak4*. (A) Alignment of wild type *pak4* sequence with mutated PCR amplicons. A pair of TALEN was designed overlapping (left arm) and downstream (right arm) the start ATG codon. The target sequences of the TAL Effector DNA-binding domains are highlighted in red. The start codon and two polymorphic nucleotides found within the right arm target are underlined. wt, wild type EK. Δ2, Δ4 and Δ5, alleles with 2-, 4- and 5-nucleotide deletion, respectively. Ins6, allele with 6-nucleotide insertion. (B) Results of immunoblotting for *pak4* protein. Total protein from 100 embryos at shield stage was collected, immunoprecipitated and analyzed using a custom-made zebrafish *pak4* anti-peptide antibody. wt, wild type EK. Δ2/Δ4, F_3_ mutants of mixed genotypes *pak4*Δ2/Δ2, *pak4*Δ4/Δ4 and *pak4*Δ2/Δ4. *pak4* protein with a predicted molecular weight of 72 kDa was detected in the wild type embryos but not in the mutants. Duplicate membrane probed with antibody to α-tubulin was used as loading control.

The TALEN reagents used in this study were designed and constructed by Cellectis (Paris, France). Briefly, candidate TALEN target sequences in the zebrafish *pak4* gene were identified (TALEN Hit Search Report), and from these we selected one immediately downstream from the start methionine codon, in order to achieve maximal disruption of the *pak4* coding sequence (see Figure 1A for positions and target sequences). TALEN expression constructs were generated in the pTAL.T7 vector backbone using the “Unit Assembly” method. Sequences of repeat-variable di-residues (RVDs) in the TAL Effector DNA-binding domains were NN-NG-NG-HD-NI-NN-HD-NI-NI-NN-NI-NI-NN-NI-NI-NG for the TALEN left arm and NG-HD-NN-NI-NI-NN-NN-NG-NN-HD-NG-NN-NI-NN-NI-NG for the TALEN right arm. Both TALENs had nuclear localization signal sequences at the N-termini. Initial validation test for cleavage activity were performed by using the single strand annealing (SSA) assay on episomal target sequences in yeast by Cellectis.

The TALEN arms were subcloned into the pCS2 vector for expression in zebrafish embryos. TALEN RNAs were synthesized in vitro by using a mMachine mMessage Transcription Kit (Ambion, USA) and injected into 1-cell stage embryos. Total protein at shield stage was then extracted for Western blot analysis to confirm successful translation (data not shown). TALEN mRNAs (25, 50, 125 and 250 pg of each arm) were injected at 1-cell stage. To detect indel mutations in the TALEN target region, High Resolution Melting Curve Analysis (HRMA) was performed. Crude extracts of genomic DNA were prepared from zebrafish embryos at 24 hpf by alkaline hydrolysis in 20 µl 50 mM NaOH at 95°C for 20 minutes followed by neutralization with 200 µl 10 mM Tris-HCl. 1 µl of this crude extract was used as template for subsequent PCR. A 106-bp amplicon flanking the TALEN target region was amplified with primer pair F13 and R17 using KAPA HRM FAST PCR Kit (KAPA BIOSYSTEMS, USA) in an Eco Real-Time PCR machine (Illumina, USA) according to the manufacturers’ instructions. The reaction profile consisted of polymerase activation at 95°C for 3 min, followed by 50 cycles of denaturation at 95°C for 5 sec and a combined annealing-extension step at 63°C for 30 sec. This was immediately followed by the HRMA profile program: the reaction mix was incubated at 94°C for 30 sec, 40°C for 30 sec and then the temperature gradually increased to 94°C. Double-stranded DNA content was monitored continuously via EvaGreen dye fluorescence. The melting curve profile of each sample was compared to that of control zebrafish EK DNA. When a significant deflection of the melting curve was observed, indicating heterogeneity in the sample, an extended PCR amplicon (primers F14 and R3) was cloned into the pGEM-T Easy vector (Promega) according to the manufacturer’s instructions, and sequenced (Macrogen, USA). The TALEN dosage showing the highest indel mutation efficiency, and less than 50% morphological toxicity at 24 hpf, was used for subsequent injection to generate TALEN mutant fish.

### Genotype Analysis

Genomic DNA from tail fin biopsies or whole embryos was prepared by the hot alkaline lysis method described above. Wild type- and mutation-specific forward primers (wt-F, Δ2-F and Δ4-F; see Table 1 for sequences) were designed at the mutation site and used for PCR with reverse primer R3. Longer amplicons (primers F14 and R3) were cloned and sequenced for verification.

**Table 1:** PCR Primers.

Name	Sequences (5′ - 3′)
F13	TTGCTTCTAGGTCTGATGCC
R17	ATCAGTGTGGACACGATGCTC
F1	TCATGAGGGCTTCACTCACTGA
R2	CTCTCGAGTCATCTCATGCGGTTTTGTCTC
R3	CAGTAGTGATGACTGTGGCATC
F17	TACGCGCACTTAGCCCGTT
MO1F	ACTTCTCACTACAGGTCTGATGCCC
MO3R	CTTCTGCCAGAGTCACTTTCAGAG
F7	TCTCACACGAGCAGTTCAGA
F2	GAGAATTCTCTGCTGCAGCCATGTTCAGCA
F14	TAATGACCCTGCTCCTTGG
wt-F	CAGCAAGAAGAAAAAGCAACG
Δ2-F	CAGCAAGAAGAAAAAGCAACA
Δ4-F	CAGCAAGAAGAAAAAGCAACC
β-actin-F	GAGGAGCACCCCGTCCTGCTCAC
β-actin-R	GATGGCTGGAACAGGGCCTCTG

### Whole Mount In Situ Hybridization

In situ hybridization of whole mount embryos at 7-somite and 24 hpf was carried out according to published procedures [15]. Antisense digoxigenin (DIG)-labeled riboprobes were synthesized using the DIG RNA Labeling kit (Roche) according to the manufacturer’s instructions.

### RNA Isolation and RT-PCR

Total RNA from whole embryos was extracted using guanidinium thiocyanate and phenol/chloroform procedures as previously published [16]. Total cDNA was synthesized by using the Superscript III Reverse Transcriptase (Life Technologies) and oligo (dT)_20_ primer according to the manufacturer’s instructions. Gene-specific primers were designed and used in PCR detection and amplification of *pak4* or *β-actin* mRNA target regions (see Table 1 for primer sequences).

### Antisense Morpholino (MO) Injection

A cocktail containing two splice-blocking (1.5 ng each) and one translation-blocking (3 ng) MOs targeting zebrafish *pak4* was injected into 1- or 2- cell stage zebrafish embryos as previously described [8]. (Note that corrected MO sequences have been reported as a corrigendum to the original article. The correct sequences are: MO-1, 5′-GCT TTA TAT TCT GAT TAC CTG ACC G-3′; MO-2, 5′-GTT CTC CCA GAG TCA CTT TCA GAG C-3′; MO-3, 5′-GGG CAT CAG ACC TGT AGT GAG AAG T-3′). A standard control MO (5′-CCT CTT ACC TCA GTT ACA ATT TAT A-3′) as well as the *pak4*-specific MOs were purchased from Gene Tools and prepared for injection according to the manufacturer’s instructions.

### Cloning of PAK4-GFP Fusion Constructs

Forward and reverse primers F1 and R2, annealing to 5′-UTR and translation stop codon of zebrafish *pak4*, respectively, were used to amplify *pak4* cDNA by RT-PCR using RNA extracted from whole embryos at 60% epiboly from control EK, *pak4*Δ2/Δ2 and *pak4*Δ4/Δ4 mutant fish. PCR products were cloned upstream of eGFP, in frame, in pCS2 vector using the Gibson Assembly kit (New England Biolabs, USA) to generate mRNAs encoding PAK4-eGFP fusion proteins. All constructs were verified by DNA sequence analysis.

### Immunoprecipitation and Western Blot Analysis

A custom-ordered anti-PAK4 peptides antibody raised in rabbit was used in immunoprecipitation and Western blotting as previously described [8]. A mouse monoclonal anti-GFP antibody (SC-9996, Santa Cruz, USA) was used for detecting enhanced GFP fusions of PAK4 in Western blotting.

### Microscopy

Live and fixed embryos, for both fluorescence and bright field, were mounted in 3% methylcellulose and 100% glycerol, respectively, and imaged and documented with Leica MZ16F microscope and Leica DFC500 camera.

## Results

### Generation and Identification of *pak4* Mutant Alleles

Two TALEN arms were designed targeting exon 2 of the zebrafish *pak4* gene, centered just downstream of the ATG start codon (Figure 1A). TALEN RNAs (50 pg left +50 pg right) were injected to produce *pak4* mutant alleles as described in Materials and Methods. Bacterial clones of PCR-amplified genomic DNA isolated from pooled (5) 24 hpf TALEN-injected embryos revealed indel mutations in the target region, ranging from 1 to 9 nucleotides deletion or insertion. Sibling embryos were raised to 2 months and screened individually by HRMA on fin biopsy genomic DNA, which identified a total of 18 out of 64 fish carrying somatic indel mutations. These carriers were out-crossed with wild type EK fish and the offspring (F_1_) were raised at a reduced culturing density (6 fish per 1.8 liter) in order to accelerate growth, allowing breeding as early as 6 weeks. Genotype analysis of the 1-month-old F_1_ fish revealed an average of 10% germ-line transmission of mutant alleles, with the same mutant allele present in all individuals derived from a single G_0_ parent. A total of four mutations were identified: deletion of 2, 4 and 5 nucleotides (Δ2, Δ4 and Δ5) and insertion of 6 nucleotides (ins6; Figure 1A). The Δ2 and Δ4 mutant alleles were identified first, and for this reason we focused on these lines for subsequent analyses. The *pak4*Δ2 and *pak4*Δ4 mutant alleles both generate frame shifts at amino acid residue #9, followed by missense polypeptides of 9 and 83 amino acids, respectively, neither of which shows significant similarity to any putative protein in the NCBI database. Wild type zebrafish PAK4 is 663 amino acids, 72 kDa [8].

In the F_1_ generation we identified 7 *pak4*Δ2 heterozygous carriers, all of which turned out to be female, while only a single male was identified as carrying the *pak4*Δ4 allele. We crossed the single Δ4 male with the Δ2 females to obtain a pool of F_2_ offspring yielding the expected Mendelian proportion of 25% trans-heterozygous *pak4* mutants (*pak4*Δ2/*pak4*Δ4), determined by DNA sequence genotyping. Juvenile *pak4*Δ2/Δ4 individuals were raised and in-crossed to obtain an F_3_ generation comprising a mix of homozygous and trans-heterozygous mutant genotypes, which was used in most experiments. For brevity, we refer to these populations as “*pak4*Δ2/Δ4”.

### Characterization of the *pak4* Knockout

**Figure 2:**
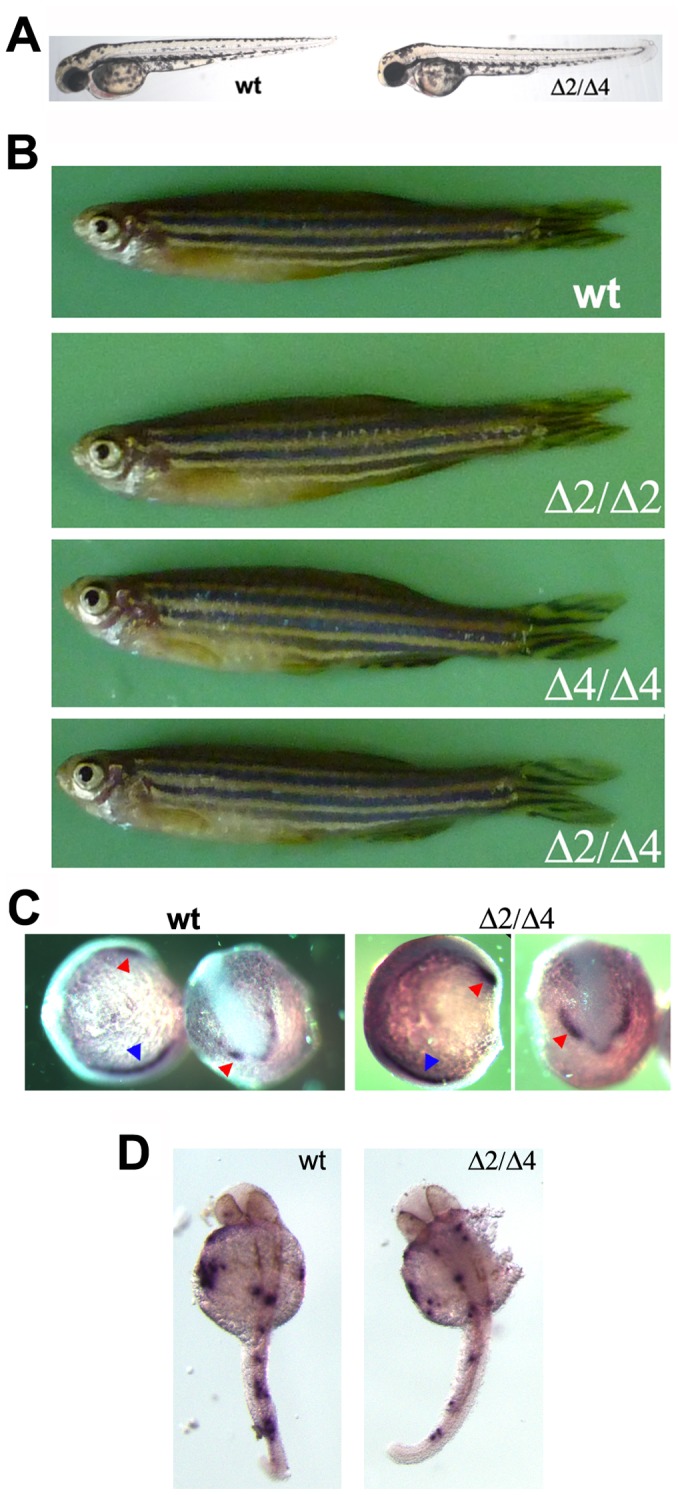
Morphological phenotype of live *pak4*Δ2/Δ4 mutants. (A) wild type and F_3_ mutant fish at 2 dpf. (B) wild type and F_3_ mutant fish at 6 months. Fish were first photographed for documentation followed by fin biopsy genotyping for confirmation. (C) Whole mount in situ hybridization of wild type and mutant embryos at 7-somite for *scl*. Lateral (left) and dorsal (right) views are shown. Red and blue arrowheads indicate anterior and posterior blood domains, respectively. (D) Whole mount in situ hybridization of wild type and mutant F_3_ embryos at 30 hpf for *mpo*. wt, wild type EK. In panels A, C and D, Δ2/Δ4 refers to the mixed population of homozygous mutant and trans-heterozygous mutant embryos. Panel B shows individuals of each defined genotype, all of which were normal and fertile.

Based on the abundance of *pak4* mRNA in zebrafish eggs and the observation that a translation-inhibiting MO, but not splice-blocking MOs, disrupted development, we concluded that *pak4* may function as a maternal-effect gene [8]. To eliminate the possibility of maternal PAK4 protein contribution to eggs from heterozygous mothers, we carried out our genetic analyses on F_3_ offspring of F_2_ compound heterozygous null individuals. To verify the loss of *pak4* expression, total protein from *pak4*Δ2/Δ4 embryos (F_3_) at shield stage was extracted for immunoprecipitation and Western blot analysis using the zebrafish PAK4 custom antibody as previously reported [8]. As shown in Figure 1B, the *pak4*Δ2/Δ4 embryos expressed no detectable PAK4 protein, indicating that both deletions represent null alleles for this gene. However, *pak4*Δ2/Δ4 embryos at 2 dpf (Figure 2A), as well as sexually mature adults (6 months old) exhibited no signs of physical abnormalities (Figure 2B). This was in contrast to the morphology of maternal-zygotic(MZ) *pak4* MO knockdown, which included reduced head and tail size, kinked body axis and lethality by 6–7 dpf [8].

In addition to physical defects, *pak4* knockdown also resulted in the inhibition of primitive myelopoiesis. Primitive erythropoiesis was unaffected, as was definitive hematopoiesis [8]. It seemed possible that transient loss of myeloid cell types might not be evident from gross morphology, so we carried out whole mount in situ hybridization of the two hematopoietic markers stem cell leukemia (*scl*) and myeloperoxidase (*mpo*) in the *pak4*Δ2/Δ4 embryos. Figure 2 shows that *scl* (C) and *mpo* (D) were expressed in the *pak4*Δ2/Δ4 embryos in normal patterns as compared to the wild type counterparts at 7-somite and 30 hpf, respectively. We conclude that unlike in *pak4* morphants, primitive myelopoiesis is normal in *pak4* null zebrafish.

**Figure 3:**
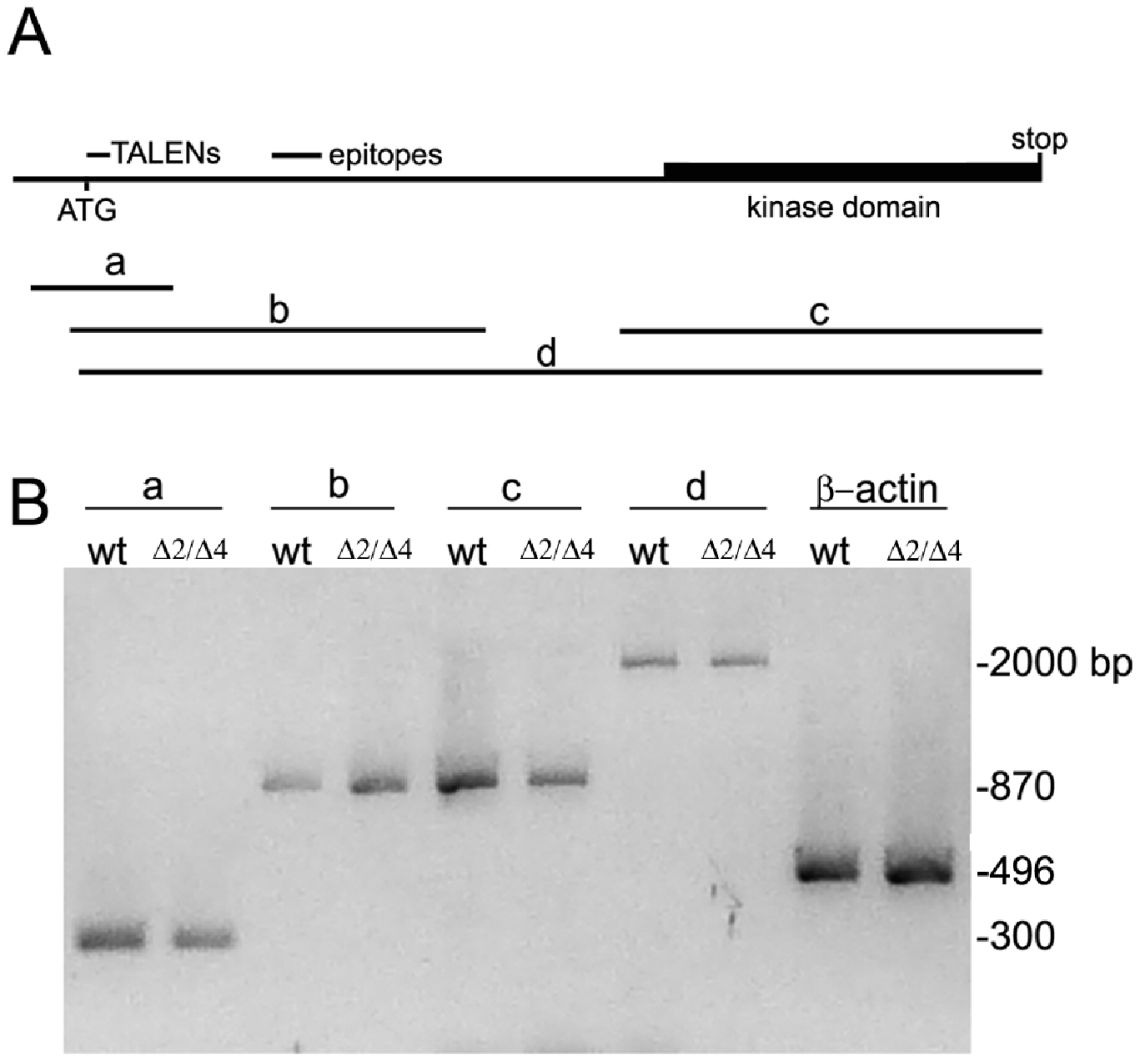
Testing for other possible forms of expressed *pak4* by RT-PCR. (A) Positions of amplicons a–d corresponding to *pak4* cDNA. a: 5′-UTR and 5′-end of coding region; primers F17 and R3. b: region amplified by primer pair (MO1F and MO3R) annealing to the target sites for the translation-blocking MO and splice MO1. c: region encoding kinase domain; primers F7 and R2. d: complete open reading frame; primers F2 and R2. TALEN mutation site and epitope site were indicated. (B) Agarose gel electrophoresis of amplicons a–d using cDNAs extracted from wild type (wt) and F_3_ *pak4* mixed null (Δ2/Δ4) embryos at shield stage as template. β-actin was used as control.

In gene-inactivation experiments it is important to verify that there exists only a single copy of the target gene in the relevant haploid genome. As previously described [8], both of the most recent versions of the EnsEMBL zebrafish genome builds (Zv8 and Zv9) were exhaustively searched for *pak4* and *pak4*-like sequence using different BLAST search engines. Similar searches were repeated in the present study with the same result, i.e. only a single copy of *pak4* could be identified in the sequenced zebrafish genome (ENSDARG00000018110; Zv9). We have also searched zebrafish cDNA/mRNA and expressed sequence tag (EST) databases, and again only a single *pak4* transcript was identified. All other sequence matches obtained from either genomic or mRNA databases corresponded to other PAK family members: *pak1a, pak1b, pak2a, pak2b, pak5, pak6a* or *pak6b* (data not shown). Absence from sequence databases is strong evidence, but is not proof, that an additional copy or copies of the *pak4* gene do not exist. We further investigated this question by performing RT-PCR using 4 primer combinations annealing to 5′-untranslated and coding regions of *pak4*. Using total RNA isolated from both *pak4*Δ2/Δ4 and wild type EK embryos at shield stage as templates, an identical pattern was obtained, with only a single product evident for each primer pair (Figure 3A, B). Cloning and sequencing of the PCR products gave only one result for each amplicon, in all cases matching perfectly to the database *pak4* version, with the exception of the 2- and 4-base deletions in RNA isolated from the mutants. Similar results were obtained at reduced annealing stringency (50°C; data not shown). In this experiment, amplicon b is especially important, as the primer targets correspond to the MO sites. Thus a hypothetical alternatively spliced mRNA that somehow bypassed the frame shift mutations would still have to include these targets sequences, since the MO cocktail was shown to block expression of the protein [8]. We conclude from these results that there is only a single *pak4* gene in the zebrafish genome, without alternative splicing within the open reading frame, and therefore neither gene duplication nor splice variants can account for the discrepancy between loss of function experiments with MOs and TALENs.

**Figure 4:**
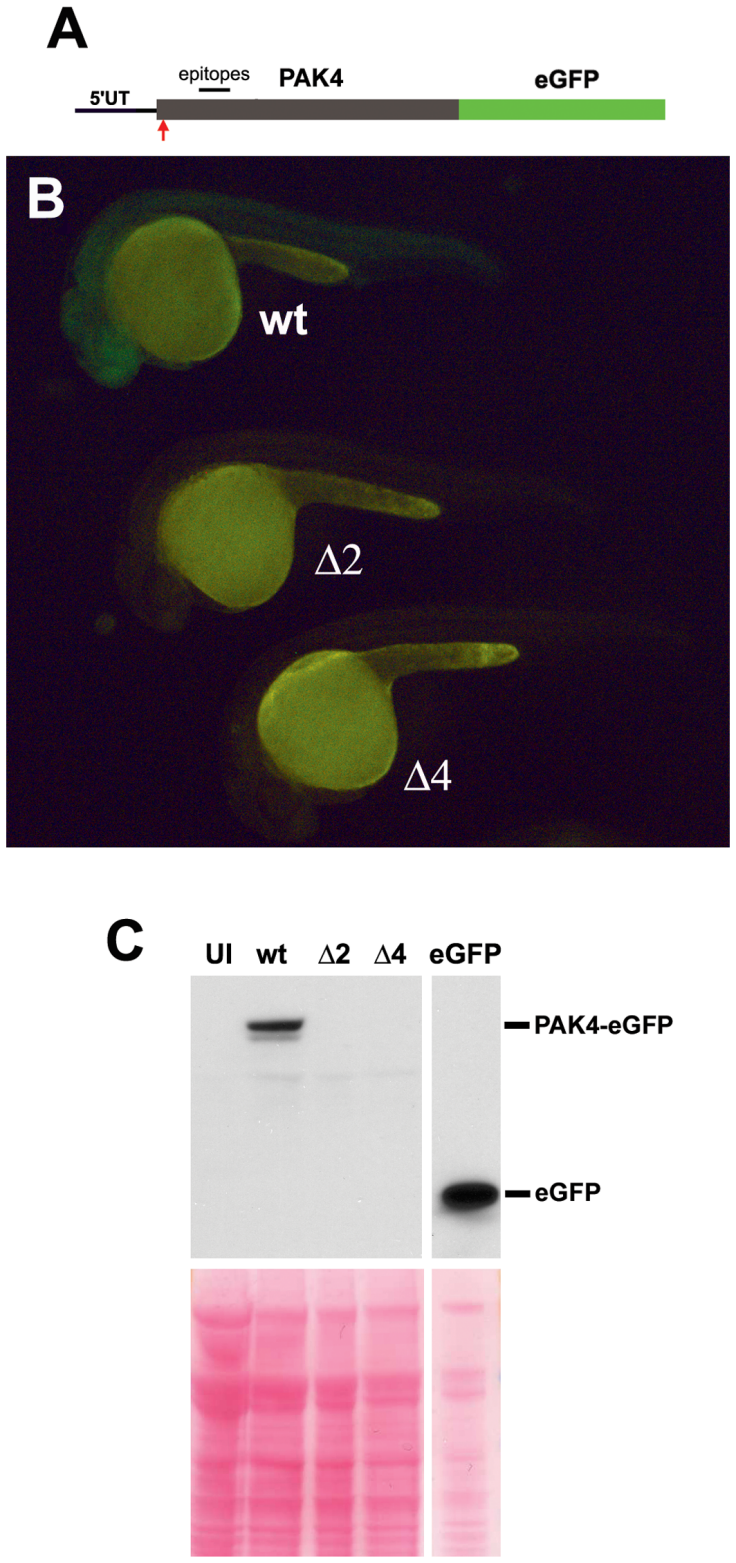
Testing for possible alternative translational initiation downstream from the mutation site. (A) Construction of the PAK4-GFP fusions. The 5′-UTR and open reading frame of *pak4* were cloned upstream of an eGFP cassette. The TALEN mutation site is indicated by the red arrow. (B) Expression of the fusions in zebrafish embryos. PAK4-eGFP RNAs were synthesized by in vitro transcription and injected into one-cell stage embryos. Green fluorescence was monitored at 24 hpf. Only the wild type construct exhibited fluorescence. (C) Western blot analysis of PAK4-eGFP RNA-injected embryos. Embryos at 24 hpf were dechorionated and used for total protein extraction and Western analysis with a GFP-specific antibody. eGFP RNA was injected into embryos as a positive control. UI, uninjected wild type EK. wt, wild type PAK4-eGFP. Δ2, PAK4Δ2-eGFP. Δ4, PAK4Δ4-eGFP. Ponceau Red staining of the Western blot membrane is shown as a loading control.

A more remote possibility we considered is that, as a result of the introduced deletions, translation of the mutant *pak4* mRNA might initiate downstream from the N-terminal methionine codon used in wild type individuals, conceivably resulting in a truncated but partially functional protein. There are three ATG codons between the deletion site and the beginning of the kinase domain (which would presumably be needed for at least some *pak4* functions). The first (amino acid 91) is in a Kozak initiation consensus (ACCATG; Kozak, 1986 [17]), but is upstream from the epitope site, and would thus result in 64-kDa protein that would have been detected by the IP-Western experiment (Figure 1B). The other two do not match the Kozak consensus (amino acid 345, CCCATG and amino acid 382, CAGATG), making this hypothesis unlikely. However, as a direct test, enhanced GPF fusions of wild type PAK4 (PAK4wt), PAK4Δ2 and PAK4Δ4 were cloned and expressed in fish embryos (Figure 4A). As shown in Figure 4B, green fluorescence was detected in the PAK4wt-eGFP RNA-injected embryos at 24 hpf while no fluorescence was found in either PAK4Δ2-eGFP or PAK4Δ4-eGFP samples. This was confirmed by Western blot analysis of the injected embryos using anti-GFP antibody (Figure 4C). These results indicate that the Δ2 and Δ4 mutations prevent expression of the downstream *pak4* reading frame.

**Figure 5:**
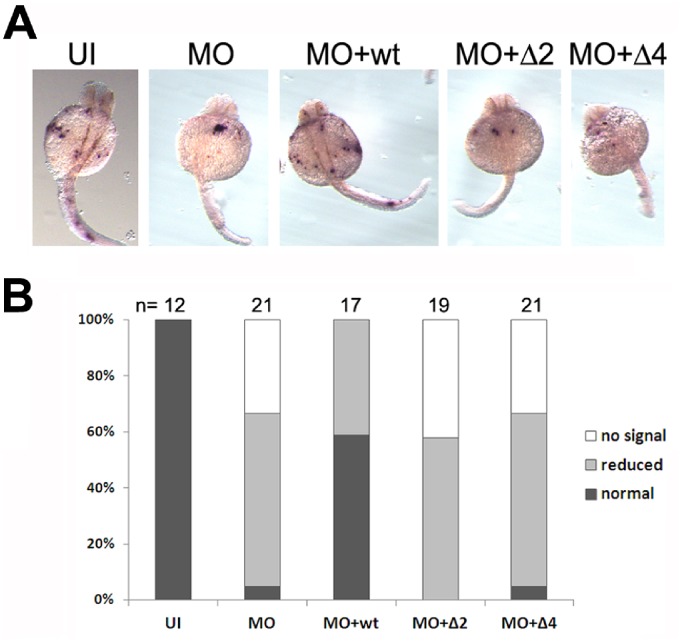
Testing the ability of mutant RNAs to rescue the MO knockdown phenotype. (A) Whole mount in situ hybridization of wild type and mutant embryos at 30 hpf for *mpo*. (B) MZ*pak4* knockdown-rescue data. Percentages of embryos showing undetectable, reduced or normal staining as compared to control for *mpo* expression at 30 hpf in (A). UI, uninjected wild type EK. MO, 6 ng *pak4* MO cocktail. MO+wt, 6 ng *pak4* MO cocktail plus 800 pg wild type PAK4-eGFP RNA. MO+Δ2, 6 ng *pak4* MO cocktail plus 800 pg PAK4Δ2-eGFP RNA. MO+Δ4, 6 ng *pak4* MO cocktail plus 800 pg PAK4Δ4-eGFP RNA. Only the wild type *pak4* RNA resulted in significant rescue of *mpo* expression.

Finally, we asked if zebrafish *pak4* mRNA might have a function in addition to serving as the template for *pak4* protein synthesis, for example, some regulatory activity residing within the open reading frame that could rescue the MO phenotype, that might be inhibited by the MOs used in our knockdown experiments. Such a regulatory function has been reported for the *squint* (*sqt*) transcript in zebrafish, in which elements residing in the 5′- and 3′- non-coding regions cooperate in an RNA-dependent and MO-sensitive scaffolding function required for dorsoventral axis specification [3]. To address this possibility for *pak4*, the RNAs for the wild type and mutant PAK4-eGFP fusions were co-injected with the MO cocktail in an attempt to rescue the knockdown phenotypes. Myeloperoxidase (*mpo*), a marker gene for normal granulocytes, was used as an indicator for myelopoiesis. As shown in Figure 5A and B, morphants expressed a reduced level of *mpo* (MO) as we reported previously [8]. Co-injection of wild type *pak4* RNA (MO+wt) rescued *mpo* expression, whereas the mutant forms, PAK4Δ2-eGFP (MO+Δ2) and PAK4Δ4-eGFP (MO+Δ4), did not. It is therefore unlikely that the discrepancy between *pak4* MO knockdown and gene inactivation by TALENs can be explained by non-coding RNA function. We conclude that the MOs used in these and previous experiments result in a highly specific but nevertheless spurious phenotype that is both independent of p53-mediated apoptosis [8] and reversible by co-injection of *pak4* mRNA.

**Figure 6:**
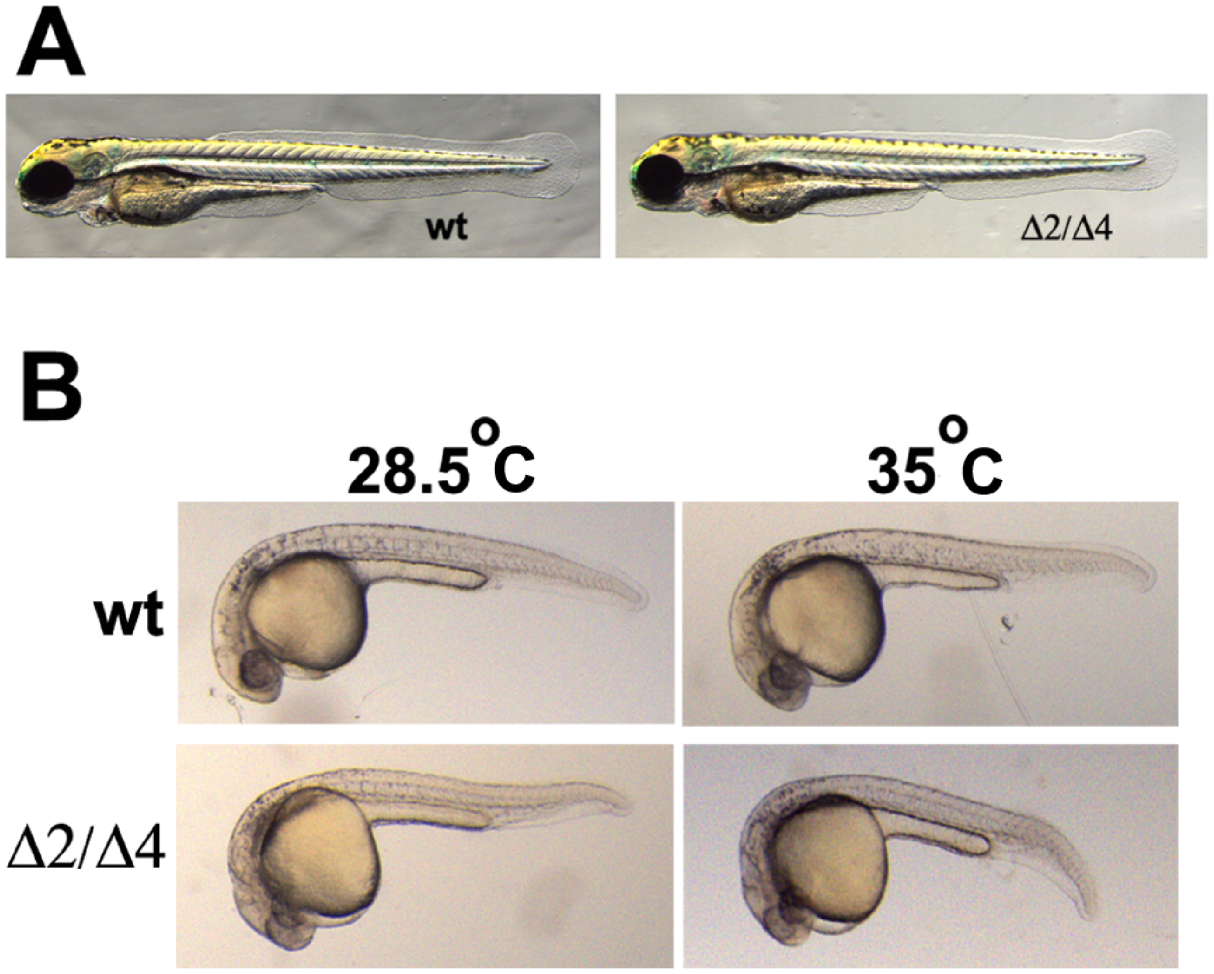
Testing for stress contribution to the *pak4* MO phenotype. (A) Injection of standard control MO (6 ng) into wild type and *pak4* mutant embryos. Live embryos at 3 dpf were anesthetized and photographed. Images shown are representatives of 92 out of 98 for wild type and 17 out of 17 for mutant. (B) Heat exposure of embryos. Wild type and F_3_ mutants were exposed to 35°C from blastula stage to 24 hpf. Control samples (28.5°C) were incubated for 5 hours longer than the heated embryos in order to compensate for different developmental rates.

What could be the basis of such an effect? One possibility is that certain MOs might induce some form of stress on embryos that could conceivably, in concert with gene-specific knockdown, result in a phenotype that might not appear in mutant embryos, since they would lack the second hypothetical stress from the MOs. We attempted to test this notion by subjecting the *pak4* mutant embryos to conditions that might lead to such a stress. First, we injected “standard control” MO (Gene Tools, USA) into wild type and mutant embryos (*pak4*Δ2/Δ4, F_3_) at the 1-cell stage, at the same dose as used for the *pak4* MO cocktail (6 ng). As shown in Figure 6A, this had no effect: both sets of embryos developed normally into feeding larvae. Second, we tested thermal stress. Zebrafish embryos develop normally between 23°C and 33°C. The recommended optimal incubation temperature is 28.5°C [14]. Exposure to elevated temperature results in the induction of heat shock chaperone proteins [18], leading to increased mortality and developmental abnormalities. Wild type and mutant embryos (Δ2/Δ4, F_3_) were incubated at 35°C from blastula stage to 24 hpf. The mutant samples exposed to high temperature developed more rapidly, but had no observable morphological phenotype at 24 hpf, compared to siblings raised at 28.5°C (Figure 6B). Identical results were obtained with wild type embryos. By 3 dpf, 25–30% of both mutant and wild type embryos receiving the 35°C treatment showed abnormalities, such as curled tail, short body axis and edema (data not shown) indicating that thermal stress did in fact disrupt development. Thus neither MO injection *per se*, nor heat stress, was able to elicit the MO knockdown phenotype in *pak4* mutant embryos. It is possible that some other form of stress, such as impurities in the original MO preparations, might have such an effect, but it is not obvious how this hypothesis could be tested.

## Discussion

Loss of *pak4* in mouse by gene targeting resulted in an embryonic lethal phenotype with multiple tissue defects including placental and epiblast vasculature, myocardial walls, spinal cord motor neurons and interneurons, caudal neural tube [12], [13]. It is surprising that in zebrafish this gene appears to be dispensable. It is reasonably clear that the locus we have studied in fish is the closest homolog to the mammalian *pak4* [8], but are these genes actually functional homologs? The genetics would seem to indicate that they are not. If there is an essential function in zebrafish that correspond to what PAK4 does in mammals, it must be performed by some other gene, or genes. It may be that PAK4, and the other Group II PAK proteins (5, 6a, 6b) have evolved different functions, or even lost essential functions altogether, during the mammalian-teleost divergence period. Such a notion is relevant to the practice of using zebrafish as a model system for the functional study of genes identified by mammalian genetics. As more loss-of-function data become available from gene targeting in fish, it will be interesting to see how common it is to find functional divergence compared to mammals, and if this might be predicted in some way *a priori.*

MOs have been in widespread use for the identification of primary gene function in zebrafish, *Xenopus* as well as other organisms [19], [20]. While this approach affords rapid and relatively inexpensive assays, it comes with serious disadvantages that are becoming increasingly evident. Off-target effects on pro-apoptotic genes have been widely reported and reviewed [6], [7]. Many studies have incorporated controls using MOs targeting the pro-apoptotic transcription factor p53 in an attempt to exclude such artifacts. Additional controls have included the use of multiple MOs targeting the same mRNA, or using injection of MO-resistant modifications of the targeted mRNA to achieve a phenotypic rescue [5].

However, as gene targeting methods have become established in zebrafish, some MO-induced phenotypes have been confirmed by loss of function alleles. For example AHR2 [21], [22], [23], WASp [24] and cas [25], [26] mutant zebrafish closely mirror the MO phenotypes. However, other examples are beginning to emerge in which MO-induced phenotypes have been validated by stringent controls, but nevertheless do not coincide with stable loss-of-function genetic mutations. The first such report concerned *Fmr1*, the zebrafish homolog of FMR1, a highly-conserved gene that is mutated in human Fragile X Syndrome. Tucker et al. [27] used MOs to knock down *Fmr1*, reporting neuronal and craniofacial abnormalities, both of which could be rescued in RNA injection controls. Subsequently, den Broeder et al. [28] reported on two zebrafish lines independently isolated from a TILLING screen with inactivating mutations in *Fmr1*. Both lines yielded normal, fertile progeny, in particular lacking any sign of the defects noted in the earlier study using MOs. A second example came from analysis of *barx1*, a homeodomain gene homologous to the human BARX1 locus. Sperber and Dawid [29] reported MO knockdown of this gene resulted in reduced chondrogenesis in pharyngeal arch derivatives. Again, this phenotype could be rescued by co-injection of suitably modified *barx1* mRNA. From another TILLING screen, Nichols et al. [30] identified two alleles with premature stop codons within the homeobox domain and thus likely to result in loss of function. These lines showed a number of defects in craniofacial morphology, but had none of the abnormalities reported with the MO knockdowns. A third case is the novel gene *inka*, which we identified using *Xenopus* and have also studied in mouse and zebrafish. MO knockdowns in frog and fish both resulted in severe craniofacial dysmorphology. These experiments were controlled by the use of multiple MOs, and parallel analysis in both species; mRNA injection controls were infeasible due to the highly disruptive nature of *inka* overexpression [31]. However, targeting the *inka* gene in mouse resulted in normal, fertile *inka* null adults [32]. Furthermore, *inka* null alleles in zebrafish have been identified by the TILLING procedure. Homozygous *inka* null zebrafish are, like the mouse, apparently normal and fertile (unpublished data). None of the TILLING-based studies carried out extensive tests for alternate expression modes that might account for the difference in phenotype compared to MOs, however the most likely interpretation in all three cases is that the MOs induced phenotypes unrelated to the target genes. It is interesting to note that craniofacial defects were prominent in all of these examples.

We have now described yet another example in which a MO phenotype, validated in this case by RNA rescue controls, fails to correspond to the phenotype of stable genetic mutations. In addition to the defects in myelopoiesis, we also observed other abnormalities in *pak4* morphants, including somite morphology, which like the myelopoiesis defect was not p53-dependent and could be rescued with *pak4* mRNA, and also defects in craniofacial cartilage, which did show a response to p53 inhibition ([8] and unpublished observations). In the *Fmr1* and *barx1* studies noted above, p53-dependence was not investigated, so it is possible that some aspects of the knockdown phenotypes might have been promoted by apoptosis. There have been anti-apoptotic functions attributed to *pak4* [33], [34], [35], [36], so it is conceivable that the p53-independent effects we observed could result from apoptosis mediated by other pathways nevertheless responsive to *pak4* overexpression. Even if such a scenario could be supported, the fact remains, however, that mutating the *pak4* gene did not elicit any of the defects found with *pak4* MOs.

In conclusion, MOs can be useful in confirming the identity of genes responsible for mutant phenotypes, or other experiments in which the loss of function phenotype has been established independently. However, when these agents are used as a primary tool to determine gene function, extensive controls are essential. Ideally, these should include both rescue by mRNA injection, and the demonstration that non-overlapping MOs yield identical phenotypes. To date, few reports based on MO experiments have met these criteria. Hence, it is likely that additional instances of incongruity between MO and genetic mutant phenotypes will emerge.
